# Intraspecific Variation of Phytochemicals, Antioxidant, and Antibacterial Activities of Different Solvent Extracts of *Albizia coriaria* Leaves from Some Agroecological Zones of Uganda

**DOI:** 10.1155/2021/2335454

**Published:** 2021-06-15

**Authors:** Timothy Omara, Ambrose K. Kiprop, Viola J. Kosgei

**Affiliations:** ^1^Department of Chemistry and Biochemistry, School of Sciences and Aerospace Studies, Moi University, Uasin Gishu County, P.O. Box 3900, Eldoret, Kenya; ^2^Africa Center of Excellence II in Phytochemicals, Textile and Renewable Energy (ACE II PTRE), Moi University, Uasin Gishu County, P.O. Box 3900, Eldoret, Kenya; ^3^Department of Quality Control and Quality Assurance, AgroWays Uganda Limited, Plot 34-60, Kyabazinga Way, P.O. Box 1924, Jinja, Uganda

## Abstract

*Albizia coriaria* Welw ex. Oliver is a customary African medicinal plant, which has a long history of utilization in the management of oxidative stress-induced and bacterial diseases. However, there is no report on the phytochemicals, antioxidant, and antibacterial activities of its leaves. The aim of this study was therefore to compare the phytochemicals, antioxidant, and antibacterial potential of *A. coriaria* leaves from Jinja, Kole, and Mbarara districts of Uganda. Shade-dried leaf samples were ground into powder and successively extracted with ethyl acetate, ethanol, and distilled water. Phytochemical screening indicated the presence of alkaloids, phenols, saponins, flavonoids, cardiac glycosides, tannins, and terpenes as the major secondary metabolites in the extracts. Total phenolic and flavonoid contents and total *in vitro* antioxidant activity were found to be the highest for ethanolic extracts, with the highest contents (101.72 ± 0.22 mg GAE/g DW; 13.23 ± 0.03 mg QE/g DW) and antioxidant potential (IC_50_ = 18.65 ± 0.06 mg/mL) being for leaves from Mbarara district. Antibacterial activity of the extracts determined by agar disc diffusion method revealed that ethanolic extracts had higher antibacterial activities with mean zones of inhibition of 6.00 ± 1.73 to 10.00 ± 1.73 mm, 5.00 ± 1.00 to 12.30 ± 1.53 mm, 17.00 ± 0.00 to 25.00 ± 2.65 mm, and 9.00 ± 1.73 to 16.00 ± 1.73 mm for *Escherichia coli*, *Staphylococcus aureus*, *Pseudomonas aeruginosa*, and *Salmonella typhi*, respectively. Ethyl acetate extracts of *A. coriaria* leaves from Kole and Mbarara had lower antibacterial activities, while aqueous extracts and ethyl acetate extract of leaves from Jinja showed no antibacterial activity. The current study for the first time established that *A. coriaria* leaves possess therapeutic phytochemicals with significant *in vitro* antioxidant and antibacterial activities, which lend credence to their use in traditional management of oxidative stress-induced conditions and bacterial diseases in Uganda. Structural elucidation of the responsible pure compounds for the observed bioactivities as well as toxicity studies of the extracts is recommended.

## 1. Introduction

Infectious diseases caused by bacteria are one of the leading causes of preventable deaths in the world [1, 2]. Nearly 50,000 people die yearly from infectious diseases, yet many of these diseases could be prevented or cured at affordable costs [3, 4]. Other pathogenic diseases, namely, human immune virus/acquired immunodeficiency syndrome (HIV/AIDS), coronavirus disease-2019 (COVID-19), and the notorious Ebola hemorrhagic fever, have also presented serious health threats [5, 6]. Cancer on the other hand has taken another toll and the situation has been exacerbated by the prevalence of carcinogenic mycotoxins in staple foods, especially in developing countries [7]. Stomach, cervical, and liver cancers have been reported to be induced or aggravated in patients with infectious diseases [8, 9]. About 55% of the 550,000 new stomach cancer cases worldwide are attributed to infections caused by pathogenic bacteria such as *Helicobacter pylori* [8]. Bacterial diseases have also been linked to increased HIV progression in immunocompromised patients [10]. Treatment of bacterial diseases is continuously becoming difficult due to the prohibitive costs, limited access, and side effects of the effective commercial antibiotics [11]. This has been made more complicated by antimicrobial resistance as it is estimated that up to 70% of known pathogenic bacteria are resistant to at least one of the antibiotics commonly used to treat diseases caused by them [12]. The complex problem of antimicrobial resistance has emerged primarily due to the indiscriminate use of antibiotics and the reemergence of diseases caused by genetically versatile microbes [13].

On the other hand, oxidative stress plays a fundamental role in the pathophysiology of various pathological conditions including infectious and neurodegenerative diseases, atherosclerosis, cardiovascular dysfunction, drug toxicity, inflammation, carcinogenesis, and reperfusion injury [14, 15]. With their ability to scavenge free radicals (reactive oxygen and nitrogen species) in the human body, antioxidants can reduce oxidative damage by donating an electron to free radicals and converting them into harmless molecules [16]. Plant materials such as fruits, seeds, vegetables, and medicinal herbs contain free radical scavenging molecules known as phytochemicals. These include phenolic and nitrogenous compounds, vitamins, terpenoids, and other endogenous secondary metabolites which have reported antioxidant activities [17]. According to the World Health Organization (WHO), at least 80% of the world's population relies on medicinal plants for their primary health care needs [18]. These plants are used due to their availability, affordability, cultural acceptability, and the general belief that herbal medicines are more efficacious and safe than allopathic drugs [19]. It is not surprising therefore that bacterial diseases and other medical conditions are being managed using medicinal plants in many parts of the world, including Uganda [19–22].

Uganda lies in the East African botanical plate and is blessed with more than 200 species of plants from over 168 genera being utilized in traditional medicine [23]. *Albizia coriaria* Welw ex. Oliver (*A. coriaria*) is one of the treasured medicinal plants in Uganda and across Africa. This is supported by its name being available in various African languages and high frequency of citation in ethnobotanical surveys. The whole plant, leaves, stem, and root barks, roots, seeds, and flowers are used in Uganda for the treatment of cancers, malaria, coronary diseases, allergy, nausea, headaches, mental illness, diarrhea, cough (tuberculosis), typhoid, anemia, syphilis, constipation, fevers, postpartum hemorrhage, snakebites, sore throats, herpes zoster, menorrhagia, threatened abortion, skin diseases, jaundice, and steam fumigation treatments for sore eyes and as an aphrodisiac and a general tonic [10, 19, 24–31]. In other African countries, this species is used in the treatment of malaria, helminthiasis, tuberculosis, diarrhea, breast, skin, and uterine cancers, menorrhagia, hypertension, dermatological conditions, threatened abortion, venereal diseases, sore eyes, lungworms/ascaris worms (in cattle, sheep, and goats), and gastrointestinal infections and as toothbrush (*Miswak*) and mosquito repellent, that is, logs burnt with cow dung [32–48].

Despite the widely reported medicinal potential of *A. coriaria* by ethnobotanical surveys, there are few reports on the phytochemistry and bioactivity of this species [25, 36, 49]. Classical phytochemical screening of aqueous and organic extracts of *A. coriaria* stem bark indicated that the active secondary metabolites are saponins, alkaloids, flavonoids, steroids, triterpenoids, reducing sugars, flavone aglycones, volatile oils, polyuronides, glucides, sterols, coumarins, and tannins [50–55]. So far, cytotoxic oleanane-type saponins, coriariosides A and B and gummiferaoside C, acacic acid glycosides (coriariosides C, D, and E), benzyl alcohol, lupeol, lupenone, betulinic acid, acacic acid lactone, and (+)-catechin, have been isolated from the root and stem bark extracts of *A. coriaria* [56–58]. Other than antitumor, antioxidant, antigiardial, molluscicidal, antiplasmodial, and antimycoplasmal activities [39, 52, 56, 59–61], most studies on *A. coriaria* assessed the antimicrobial activity of its stem bark crude extracts [11, 26, 53, 55, 58, 62, 63]. The current study, for the first time, aimed to evaluate the antibacterial activity of *A. coriaria* leaves to verify its claimed traditional use in the treatment of bacterial diseases in Uganda [19, 31, 64, 65]. The study further performed preliminary phytochemical screening and evaluated the total phenolic content, total flavonoid content, and antioxidant activity of *A. coriaria* leaves.

## 2. Materials and Methods

### 2.1. Chemicals, Reagents, Bacterial Media, and Culture

All chemicals and reagents used in this study unless otherwise indicated were of analytical grade and were used without subjection to any further purification. Ethyl acetate, ethanol, methanol, dimethyl sulfoxide, extra pure (99.5%) gallic acid, quercetin (>99.5%), Folin–Ciocalteu reagent, ascorbic acid, 1,1-diphenyl-2-picryl-hydrazyl (DPPH) reagent, aluminium chloride, sodium carbonate, sodium hydroxide, concentrated sulfuric acid, ferric chloride, glacial acetic acid, concentrated hydrochloric acid, iodine, and potassium iodide, acetic anhydride, and chloroform were supplied by Centrihex Limited, Nairobi, Kenya. Muller Hinton agar (Oxoid, UK), Luria-Bertani agar, Miller (Sigma Aldrich, St. Louis, USA), Nutrient agar (HiMedia Laboratories Pvt. Ltd., Mumbai, India), ciprofloxacin, *Escherichia coli* ATCC 25922, *Staphylococcus aureus* ATCC 25923, *Pseudomonas aeruginosa* ATCC 27853, and *Salmonella typhi* 14028 were supplied by Kobian Kenya Limited, Nairobi, Kenya.

### 2.2. Collection, Authentication, and Preparation of Samples

**Figure 1:**
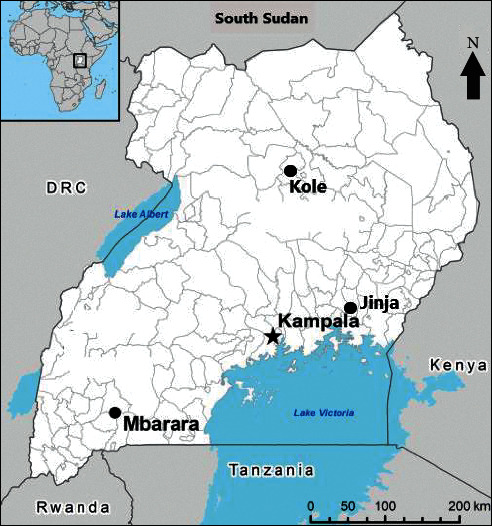
Map of Uganda showing districts where *A. coriaria* leaf samples were obtained. Inset is the location of Uganda on the African continent.

Mature *A. coriaria* leaves (5 kg) were collected from plants in their natural habitats in Jinja, Kole, and Mbarara districts of Uganda representing the South East, Mid Northern, and Southern drylands agroecological zones, respectively (Table 1; Figure 1).

The samples were collected between January 2021 and February 2021. They were identified and authenticated by Kyoshabire Medius (a taxonomist) at the Department of Botany, Natural Chemotherapeutics Research Institute, Wandegeya, Kampala, Uganda. Specimen samples with voucher numbers 50994, 50995, and 50996 were deposited at Makerere University Herbarium, Kampala, Uganda. The laboratory samples were washed under running tap water and shade-dried for two days in nearby laboratories at room temperature (25.0 ± 2.0°C) to avoid their deterioration prior to transportation for further drying. Thereafter, they were packed in clean polyethylene bags and transported to Moi University Chemistry Laboratory, Eldoret, Kenya, where they were separately air-dried under shade at room temperature (25.0 ± 2.0°C) for 3 weeks.

The dry leaves were ground into fine powder using a NutriBullet® 600 Series electric grinder (Capbran Holdings, LLC Los Angeles, USA). The method used was sequential extraction. The powders (500 g) were separately weighed using a Mettler Toledo digital analytical balance (XS204 Delta Range, Switzerland), transferred into 2000 mL conical flasks, and then macerated at room temperature for 48 hours in 1000 mL of ethyl acetate with occasional shaking [66]. The extracts were filtered through cotton wool and subsequently Whatman No. 1 filter paper, while the residues (labelled R1) were used in the subsequent extraction procedure. Residues (R1) were air-dried for 48 hours at room temperature and macerated with 1000 mL of ethanol in 2000 mL conical flasks at room temperature for 48 hours with intermittent shaking. After 48 hours, the solutions were filtered through cotton wool and then Whatman No.1 filter paper. The second set of residues (labelled R2) were kept for further extraction. Residues (R2) were air-dried for 48 hours and thereafter macerated in 1000 mL of distilled water at room temperature in 2000 mL conical flasks for 48 hours with occasional shaking. The solutions were filtered through cotton wool and Whatman No. 1 filter paper.

The organic solvent extracts were concentrated to dryness on a Hahnvapor HS-2005S vacuum rotary evaporator (Hahnshin S&T Limited, Korea) at 40°C under reduced pressure, while the aqueous extracts were lyophilized. The concentrated extracts were collected in preweighed and labelled 10 mL sample vials and weighed and their masses (in grams) were recorded. The total extractable components (percentage yield) of each solvent extract were calculated using the following equation [67]:

(1) $$\begin{array}{c} \text{percentage yield}=\left(\frac{A}{A_{0}}\right)\;\times \;100, \end{array}$$

where *A* denotes the mass of crude extract obtained after drying and *A*_0_ denotes the mass of the leaves used for extraction.

The dried extracts were transferred to a desiccator containing anhydrous sodium sulfate to remove traces of water in them. The extraction process was replicated once.

### 2.3. Preliminary Phytochemical Screening of *A. coriaria* Leaf Extracts

Dried extracts were dissolved in their respective solvents of extraction in a ratio of 1 : 10 (w/v). Standard screening procedures by Trease and Evans [68] were followed to test for the presence of alkaloids, flavonoids, cardiac glycosides, phenols, saponins, quinones, steroids, tannins, terpenes, and volatile oils in all the extracts of *A. coriaria* leaves. The results were expressed in terms of the relative abundance of the respective secondary metabolites [69, 70].

### 2.4. Determination of Total Phenolic Content, Total Flavonoid Content, and Antioxidant Activity of *A. coriaria* Leaf Extracts

#### 2.4.1. Total Phenolic Content

Total phenolic content (TPC) of the extracts was determined spectrophotometrically using the Folin–Ciocalteu method reported by Velioglu et al. [71] with some modifications. Dry crude extracts (0.01 g) of *A. coriaria* leaves were dissolved in 10 mL of distilled water. From this, 0.01 mg/mL solutions were prepared by dissolving 0.1 mL of the resultant solutions in 10 mL of distilled water. A volume of 0.5 mL of the resultant solutions was measured and transferred into vials in triplicate and mixed with 2.5 mL of Folin–Ciocalteu reagent. After 7 minutes, 2.5 mL of 6% (w/v) sodium carbonate solution was added. The solutions were incubated in the dark at room temperature (25.0 ± 2.0°C) for 30 minutes after which their absorbance was measured at 725 nm using a general-purpose (single beam) UV-Vis spectrophotometer (Beckham Coulter DU 720, Beckham Coulter Inc., USA). A calibration curve was prepared for quantitative analysis of TPC in the extracts using gallic acid as the standard. Gallic acid solutions (10, 20, 40, 60, and 80 mg/mL) were prepared. Measured 0.5 mL of the solutions was transferred into vials and given the same treatment as done for the extracts. The TPC of the extracts was determined as mg gallic acid equivalents per gram of dry weight (mg GAE/g DW) from the calibration curve.

#### 2.4.2. Total Flavonoid Content

The total flavonoid content (TFC) of the extracts was determined using the aluminium chloride colorimetric assay reported by Pękal and Pyrzynska [72] with some specific modifications [73]. Crude extracts of *A. coriaria* leaves (0.01 g) were completely dissolved in 5 mL of methanol. Standard solutions of quercetin (5, 10, 25, 50, 75, and 100 ppm) were prepared by serial dilution using methanol. Micropipetted 0.6 mL diluted standard quercetin solutions and extracts were separately mixed with 0.6 mL of 2% (w/v) methanolic aluminium chloride solution in test tubes. After mixing, the solutions were incubated for 1 hour at room temperature (25.0 ± 2.0°C). The absorbance of the reaction mixtures was measured against methanol blank at 420 nm on a UV-Vis spectrometer (Beckham Coulter DU 720, Beckham Coulter Inc., USA). A calibration curve was obtained from the quercetin standard absorbances and the TFC of the extracts was determined as mg quercetin equivalents per gram of dry weight (mg QE/g DW) of the plant extracts.

#### 2.4.3. Free Radical Scavenging Activity Using DPPH Assay

The potential of *A. coriaria* leaf extracts to directly react with and quench free radicals was evaluated using the 2,2-diphenyl-1-picryl-hydrazyl (DPPH) free radical scavenging assay as described by Khiya et al. [74] with some modifications. Briefly, dry crude extracts were dissolved in methanol to produce solutions of 10, 15, 25, 50, and 60 *μ*g/mL. Pipetted 75 *μ*L of the freshly prepared methanolic DPPH solution (1.3 mg/mL) was added to each of the test tubes containing 200 *μ*L of the extracts and incubated in the dark for 30 minutes at room temperature. Ascorbic acid was used as the reference standard but was dissolved in distilled water to make solutions with the same concentrations as that of the extracts. The absorbances of the assay mixtures were read at 517 nm on a spectrophotometer (Beckham Coulter DU 720, Beckham Coulter Inc., USA). Methanol was used as the blank for the extracts, while distilled water was used as a blank for ascorbic acid. The percentage of DPPH radical inhibition was calculated using equation (2). The half-inhibitory concentration (IC_50_) was determined from the inhibition percentage curve as a function of the extract concentrations [74].

(2) $$\begin{array}{c} \text{percentage inhibition}=\frac{A_{0}\;-\;A_{S}}{A_{o}}\times 100, \end{array}$$

where *A*_*o*_ is the absorbance of the solution containing only DPPH radical solution as a negative control and *A*_*S*_ is the absorbance of the sample solution in the presence of DPPH.

### 2.5. Antibacterial Screening of *A. coriaria* Leaf Extracts

Antibacterial activity of *A. coriaria* leaf extracts was tested against American Type Culture Collection bacterial strains of *E. coli*, *S. aureus*, *P. aeruginosa*, and *S. typhi* in the Biological Sciences Laboratory, Moi University, Kenya. Selection of the bacteria was based on the diseases for which the leaves are traditionally used, the WHO priority list, and availability [75, 76]. All materials, including bacterial media used in the assay, were autoclaved at 121°C and 15 psi pressure for 30 minutes in an All-American® Steam Sterilizer (Model No. 25X, Wisconsin Aluminium Foundry Co. Inc., USA). Bacterial inoculation was performed under a laminar flow cabinet disinfected with 70% ethanol (v/v). Ultraviolet radiation from the cabinet was used for sterilization prior to bacterial inoculations.

#### 2.5.1. Agar Disc Diffusion Assay for Antibacterial Activity Screening

Determination of antibacterial activity of the *A. coriaria* leaf extracts was achieved using the agar disc diffusion method. Test extracts (500 *µ*g/mL) were prepared using sterile dimethyl sulfoxide [61]. Four sterile paper discs (6.0 mm) were saturated with each prepared test extract and dried [70]. Test bacteria (1 × 10^8^ colony-forming units/mL) previously subcultured on nutrient broth and Luria-Bertani broth (for *S. aureus*) were aseptically inoculated onto sterile Muller Hinton broth in 90 mm Petri dishes. Using sterile forceps, extract-impregnated discs were gently placed on each plate in triangular formation. A separate agar plate was used to test the positive control (ciprofloxacin, 5 *µ*g disc) against the bacterial strains. Dimethyl sulfoxide was used as the negative control. The plates were sealed using parafilm, inverted, and incubated at 37°C for 24 hours in a thermostatically regulated bacteriological incubator. Using a metric ruler, the diameter of the zone of inhibition was measured for plates with inhibited bacterial growth after 24 hours.

#### 2.5.2. Determination of Minimum Inhibitory Concentration and Minimum Bactericidal Concentration

Minimum inhibitory concentration was determined for bacteria (*S. aureus, P. aeruginosa*, and *S. typhi*) that exhibited the highest sensitivity to ethanol extracts with inhibition diameters above 12 mm upon screening [77]. Pipetted 500 *μ*L of bacteria was serially diluted from 2-fold to 4-fold dilution in sterile Muller Hinton broth. Five hundred microliters of the test bacteria was aseptically inoculated in each of the four tubes containing the extracts of concentrations 500, 250, 125, and 62.5 *µ*g/mL prepared using dimethyl sulfoxide. Thereafter, the tubes were incubated at 37°C for 24 hours. After incubation, the tube next to the one showing no microorganism turbidity was considered as containing the minimum inhibitory concentration of the extract.

Following minimum inhibitory concentration determination using the broth dilution method, 4 mL of the test extract in order of increasing dilution was subcultured on Muller Hinton media and incubated at 37°C for 24 hours. The highest dilution which showed no growth of the bacteria was considered as the minimum bactericidal concentration.

### 2.6. Statistical Analysis

All experiments were performed in triplicate except extraction which was performed in duplicate. Quantitative data were expressed as means ± standard deviations of replicates. The means were separated by one-way analysis of variance (ANOVA). To identify the source of significant differences between means, Tukey honest significant difference (HSD) test was performed. A difference was considered statistically significant if *p* < 0.05. Correlations among TPC, TFC, and antioxidant activity of the extracts were assessed using Pearson's bivariate correlation. All statistical analyses and graphical work were done using GraphPad Prism statistical software (version 9.1.0, GraphPad Software, California, USA).

## 3. Results and Discussion

### 3.1. Extraction Yield of *A. coriaria* Leaves

**Figure 2:**
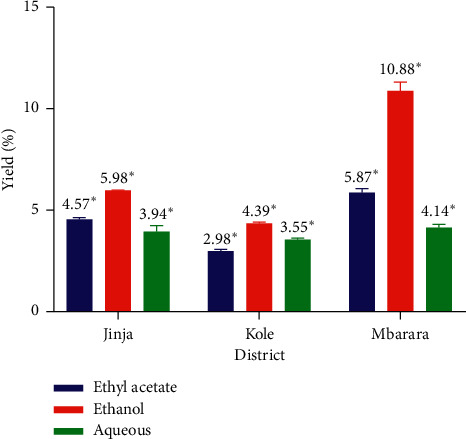
Percentage yield of the different solvent extracts of *A. coriaria* leaves. Means with asterisks are not statistically different for the different solvent extracts.

The extraction yields shown in Figure 2 were expressed as percentages of the initial mass of the dry leaf powders macerated. The dry crude organic solvent extracts were green, while aqueous extracts were brown. Maceration in ethanol gave the highest yields in comparison to ethyl acetate and distilled water with *A. coriaria* leaves from Mbarara having the highest yield of 10.88 ± 0.12%. The extract yields recorded for *A. coriaria* leaves were comparable to the yields of 8.3% and 12.7% for ethyl acetate and methanolic extracts of *A. coriaria* stem bark from Mukono district (Lake Victoria Crescent agroecological zone) of Uganda reported by Ganza [77]. However, ANOVA results indicated that the extraction yields were not significantly influenced by the extraction solvent used (*p* > 0.05). From the obtained results, it can be inferred that ethanol was a good solvent for extraction of phytochemicals in *A. coriaria* leaves compared to ethyl acetate probably because most compounds in *A. coriaria* leaves are polar. Thus, the phytochemicals were able to dissolve in the more polar ethanol than ethyl acetate used initially in the sequential extraction. Aqueous extracts had the lowest yields because most of the polar phytochemicals had already been extracted by the moderately polar ethanol before the leaf residues (R2) were macerated with distilled water.

Differences in polarities of solvents employed in serial extraction are known to play a key role in increasing the solubility of phytochemicals in plant matrices [78, 79]. Further, differences in the structure (functional groups) of phytochemicals, time, temperature, solvent concentration, and solvent polarity also influence the solubility of phytochemicals in organic or polar solvents or solvents of different polarities [67]. The three solvents used for serial exhaustive extraction in this study had different polarity indices, arranged as ethyl acetate (4.4) < ethanol (5.2) < water (9.0) [67, 80]. Therefore, the results of the current study confirmed the richness of *A. coriaria* leaves in both polar and nonpolar phytochemicals as previously indicated for its stem bark [61, 77].

Among the three districts, the yield for ethanolic extract of *A. coriaria* leaves from Mbarara was nearly two times higher than that of leaves from Jinja and Kole despite using the same extraction solvent and method (Figure 2). A similar pattern was noted for yield of ethyl acetate extract of leaves from Mbarara being almost twice that of leaves from Kole. The intraspecific variation in the yields of the extracts of *A. coriaria* leaves could be due to extrinsic factors such as differences in soil chemistry, season, topography, and climate which are known to affect the phytochemical yield of plant organs [81–84]. Mbarara receives an average rainfall of 1,200 mm per annum with two distinct rainy seasons between February and May and then September and December. The temperature fluctuates between 17°C to 30°C corresponding to a humidity range of 80–90% [85]. The topography is a mixture of fairly rolling and sharp hills and mountains, shallow valleys, and flat land lying at about 1483 mm above sea level. The soils are sandy, clay, and slightly laterite loams. The sampling area in Jinja on the other hand is on the shores of Lake Victoria (near the source of River Nile). The area has warm temperatures ranging between 23°C and 32°C and a bimodal rainfall pattern averaging 1260 mm annually [86]. The soils in this area are primarily made of granites and granitoid gneisses, with patches of shales, phyllites, and schists [86]. It is located about 1,187 m above sea level, with valleys in some parts of the district. Kole district lies in the Northern part of Uganda, at an elevation of 1,073 m [87]. It was formed out of Apac district on 1 July 2010 [88]. This area has a unimodal season with a total annual rainfall of 1,330 mm (from April to November) [89]. The dry season is from December to March and the average monthly minimum and maximum temperatures are 17°C and 29°C, respectively. Thus, leaf samples from Mbarara recorded the best yields possibly because of the good soils in the district than in the other districts [90]. The district is also the coolest of the three studied districts. Previous studies have indicated that plant materials sampled from plants growing in cooler areas (at higher altitudes) tend to give higher extraction yields than those in warm climatic conditions and lowland areas [81, 82]. In addition, the time of sampling could have led to the disparities in the yields obtained. For example, Kole district was in a complete dry season when the leaves were sampled, while Mbarara district was entering a rainy season when the samples were taken.

### 3.2. Phytochemical Screening of *A. coriaria* Leaves

Qualitative phytochemical screening of medicinal plants informs the need and choice for their elaborate phytochemical analysis and/or pharmacological evaluation [91, 92]. Classical phytochemical screening of the different solvent extracts of *A. coriaria* leaves revealed the presence of several secondary metabolites including alkaloids, flavonoids, cardiac glycosides, phenols, saponins, tannins, and terpenes as shown in Table 2. However, quinones, steroids, and volatile oils were not detected in all the extracts. Alkaloids were absent in the ethyl acetate extract of *A. coriaria* leaves from Mbarara and in ethanolic extracts from Jinja and Kole. Flavonoids were absent in the ethyl acetate extract from Jinja, while tannins were also not detected in ethanolic extracts of leaves from Jinja.

The results of the present study agreed well with previous reports on *A. coriaria* stem bark which reported the presence of tannins, saponins, alkaloids, flavonoids, terpenes, and cardiac glycosides as the major secondary metabolites [50–55, 62]. Further, the identified metabolites have been previously reported in the leaf extracts of other *Albizia* species such as *A. lebbeck* (L.) Benth [93] and *A. harveyi* [76]. The absence of alkaloids was previously reported for ethyl acetate and aqueous extracts of *A. lebbeck* leaves [93, 94] and *A. zygia* leaves [95] as well as ethanolic leaf extracts of *A. harveyi* [76]. The total absence of steroids in *A. coriaria* leaves corroborates a previous observation in which steroids were absent in ethyl acetate and aqueous extracts of *A. lebbeck* leaves [94]. Volatile oils could have been absent in the extracts because the solvents used in this study are not able to extract them from the leaves. These intraspecific variations in secondary metabolites in *A. coriaria* leaves could be due to differences in soil chemistry, rainfall, topography, and climate that affect the interaction between plants and the environment and ultimately the depot of bioactive compounds in plant leaves [96, 97].

### 3.3. Total Phenolic Content, Total Flavonoid Content, and Antioxidant Activity

#### 3.3.1. Total Phenolic Content

The TPC of *A. coriaria* extracts was determined using the Folin–Ciocalteu method. A calibration curve was prepared for quantitative analysis and the linearity for gallic acid standard was established from the range of 10 ppm to 80 ppm which was fitted on the line *y* = 0.0126*x* − 0.0107 with *R*^2^ = 0.9992. As shown in Table 3, TPC was highest for ethanol extracts, with ethanolic extracts of leaves from Mbarara showing the highest TPC of 101.72 ± 0.22 mg GAE/g DW. In comparison to extracts from Jinja and Kole, all extracts of *A. coriaria* leaves from Mbarara had the highest TPC (Table 3). This could be because Mbarara has good soils than in the other districts [90]. The district is also the coolest of the three studied districts, which could explain the higher accumulation of phenolics in the leaves. As reported by Cansev et al. [98] and Król et al. [99], cooler climates are associated with increased phenolics production, possibly for plant self-defence against environmental stress.

One-way ANOVA results showed that there were significant differences (*p* < 0.05) between the mean TPC of the different solvent extracts of *A. coriaria* leaves. Schultz et al. [61] reported that the TPC of ethanol extracts of *A. coriaria* stem bark (28.37 ± 0.34 milligram chlorogenic acid equivalent per gram of extract, mg CAE/gE) was only slightly higher than that of its ethyl acetate extract (28.36 ± 0.97 mg CAE/g E). A plausible explanation for this is that ethanol is a polar protic solvent. Thus, it extracted more polyphenols which are inherently polar through hydrogen bond formation [100, 101]. Further, water is known to extract nonactive compounds in plant matrices including proteins and sugars which do not contribute to the TPC and some biological activities of plant extracts [78].

#### 3.3.2. Total Flavonoid Content

The aluminium chloride method was used for TFC determination. This method is based on the formation of aluminium-flavonoid complexes [72]. The colorimetric assay using aluminium chloride detects flavonoids in the flavone and flavonol groups as in quercetin [102]. In this study, a calibration curve prepared using quercetin as a standard was used to quantify the TFC of the extracts. Linearity for the standard was established from 5 ppm to 100 ppm which was fitted on a straight line that gave the equation *y* = 0.0109*x* + 0.0851 with *R*^2^ = 0.9987. The mean TFC of the extracts was highest for ethanolic extracts, with ethanol extract of leaves from Mbarara district giving the highest mean of 13.23 ± 0.03 mg QE/g DW (Table 4). This could have been because ethanol with higher polarity than ethyl acetate was able to extract much of the flavonoids which are relatively polar [67]. This indicates that the *A. coriaria* leaves contain more flavonoid heterosides than aglycones [101].

In addition, all extracts from Mbarara district had the highest TFC compared to the extracts from Jinja and Kole districts (Table 4). As explained for the yields and TPC, this could be because Mbarara is the coolest of the studied districts and the variations in the seasons in which the sampling was done [103]. One-way analysis of variance performed indicated that there were significant differences (*p* < 0.05) between the mean TFC of the different solvent extracts. Further, the mean TFC obtained for the extracts was lower than their corresponding mean TPC values. High TPC of plant extracts than TFC supports the chemistry that most flavonoids are also phenolics [104]. Overall, the recovery of phytochemicals from plant matrices is influenced by the dielectric constant and structure of organic solvents as well as the chemical properties of the phytochemicals [101]. This explains the variations observed in the TPC and TFC of *A. coriaria* leaf extracts from the same district extracted using the three different solvents. The low levels of total flavonoids in the extracts also agreed with the phytochemical screening results (Table 2) which indicated that there were only traces of flavonoids in ethyl acetate and aqueous extracts. This observation indicates that most of the flavonoids in *A. coriaria* leaves are polar as they were able to get extracted more by ethanol than ethyl acetate. The lower TFC of aqueous extracts could be because water extracts even nonactive compounds in plant materials including proteins and sugars which do not contribute to the TFC of plant extracts [78].

#### 3.3.3. Free Radical Scavenging Activity Using DPPH Assay

Antioxidant activity of phytochemical compounds is ascribed to their reduction-oxidation properties which enables them to function as hydrogen donators (reducing agents), metal chelators, or singlet oxygen quenchers [105]. The DPPH test used in this study measures the hydrogen atom or electron-donating capacity of alcoholic plant extracts to the stable radical DPPH formed in solution. Antioxidants in natural products react with DPPH through hydrogen atom transfer (HAT) and/or multistep reactions (single electron transfer and sequential proton loss electron transfer) or a synergistic combination of these mechanisms [105]. In nonaqueous media, single electron transfer and sequential proton loss electron transfer mechanisms take precedence due to the enhanced chemical reactivity of the organic solvents to form hydrogen bonds with the antioxidants [105].

The results of *in vitro* antioxidant activity assay (Table 5) indicated that ethanolic extracts had the lowest IC_50_ values. However, these were higher than that of ascorbic acid (0.17 ± 0.01 mg/mL). There were significant differences (*p* < 0.05) between the antioxidant activity of ethyl acetate, ethanol, and aqueous extracts of *A. coriaria* leaves. These differences could be explained by the differences in the phytochemical composition of the different extracts, possibly due to geographical, soil, climate, and genetic variations experienced in the different agroecological zones [82, 96, 97]. Further, it was noted that ethanolic extracts had the highest antioxidant activity. This could be because most phenolic compounds that account for the antioxidant activity of plant extracts possess polar functional groups and are therefore easily dissolved in polar protic solvents like ethanol through hydrogen bond formation [106]. The results of antioxidant activity in this study were higher than those reported for ethyl acetate and ethanolic extracts of *A. coriaria* stem bark sampled from Mpigi district (Lake Victoria Crescent agroecological zone). The extracts had half effective concentrations (EC_50_) of 0.02298 ± 0.00247 mg/mL and 0.01839 ± 0.00223 mg/mL, respectively [61]. The results obtained are similar to those of Do et al. [107] in which ethanolic extracts of *Limnophila aromatica* aerial parts had the highest antioxidant activity and aqueous extracts had the lowest antioxidant activity. The lower antioxidant activity of aqueous extracts could be explained by the fact that water extracts even nonactive compounds in plant materials including proteins and sugars which do not contribute to the antioxidant activity of plant extracts [78].

#### 3.3.4. Correlation between Total Phenolic Content, Total Flavonoid Content, and Antioxidant Activity

A strong positive correlation (*R* = 0.898, *p*=0.001) between TPC and TFC of the extracts was observed. This is usually expected because total phenolic compounds comprise both flavonoids and nonflavonoid polyphenols. Thus, a strong positive correlation indicates that TFC of the extracts contributes significantly to the TPC of the extracts [108]. Pearson's bivariate correlation coefficient between TPC and antioxidant activity of the extracts revealed that TPC exhibited a high negative correlation with the IC_50_ values obtained in DPPH assay (*R* = −0.831, *p*=0.006). In the same way, TFC was negatively and highly correlated (*R* = −0.755, *p*=0.019) with IC_50_ values obtained in DPPH assay. Schultz et al. [61] observed a poor correlation between TPC and the corresponding antioxidant activity (EC_50_ values obtained using DPPH assay) of ethyl acetate and ethanolic extracts of *A. coriaria* stem bark. The negative correlation between DPPH radical quenching activity, TPC, and TFC is because the radical content decreases as the activity of the extract increases. This therefore implies that the total phenolic or total flavonoid compounds may play a significant role in increasing DPPH radical scavenging activity of the extracts [108].

### 3.4. Antibacterial Activity of *A. coriaria* Leaves

#### 3.4.1. Antibacterial Screening Results

In this study, the antibacterial activity of crude extracts of *A. coriaria* leaves from different agroecological zones of Uganda was assessed using the agar disc diffusion method. All the ethanol extracts and ethyl acetate extracts of leaves from Kole and Mbarara had an inhibitory effect on the growth of the tested microorganisms, while the aqueous extracts did not have any antibacterial activity (Table 6). The activity was higher for ethanolic extracts than ethyl acetate extracts. The negative control (DMSO) showed no inhibitory activity on the tested bacteria, while ethanol extracts had comparable antibacterial activity to ciprofloxacin. For example, ethanol extract from Kole had a marked antibacterial activity against *S. typhi* with an inhibition diameter of 16.00 ± 1.73 mm which was comparable to that of ciprofloxacin (inhibition diameter of 20.00 ± 1.53 mm). Only ethanolic extracts had inhibition zones greater than 12 mm against some of the targeted bacteria after screening, that is, Jinja ethanol extract against *P*. *aeruginosa*, Kole ethanol extract against *P*. *aeruginosa* and *S. typhi*, and Mbarara ethanol extract against *S. aureus* and *P*. *aeruginosa*. Thus, the bacteria (except *S. aureus*) were regarded as susceptible to the ethanolic extracts as the respective zones of inhibition were within the range for standard antibiotics such as ampicillin, doxycycline, and tetracycline as per Clinical and Laboratory Standards Institute Interpretive Criteria [109].

The antibacterial activity results confirmed that more bioactive compounds were extracted by ethanol than ethyl acetate and distilled water. This observation is in agreement with a report by Schultz et al. [61] who found that ethanolic extracts of *A. coriaria* stem bark harvested from Mpigi district (Lake Victoria Crescent agroecological zone) of Uganda were more active against pathogenic bacteria than the ethyl acetate extract. This could have been because the phytochemicals in *A. coriaria* leaves are more soluble in ethanol than ethyl acetate and were extracted by ethanol before they were macerated in distilled water. This observation is contrary to the results of Byamukama et al. [58] in which ethyl acetate extracts of *A. coriaria* stem bark from Mukono district (Lake Victoria Crescent agroecological zone) of Uganda had the highest antibacterial activity compared to methanolic and aqueous extracts. The complete inactivity of aqueous extracts of *A. coriaria* leaves was previously reported for *A. coriaria* stem bark serially extracted with ethyl acetate, methanol, and distilled water [58]. The inactivity of nonpolar solvent extracts of this species was also reported where hexane extracts of *A. coriaria* stem bark were not active on *S. aureus*, *E. coli* isolate, clinical *S. aureus*, and *E. coli* ATCC 25922 [53]. Similarly, aqueous and ether extracts of *A. coriaria* stem bark were not active when tested *in vitro* against *E. coli* and *S. typhi* [62]. Ethanolic extracts of *A. coriaria* stem bark at 256 *μ*g/mL were recently indicated to have no inhibitory effect on *S. aureus* UAMS-1 among other multidrug resistant bacteria [11].

The identified secondary metabolites in the ethanol and ethyl acetate extracts could be responsible for the observed antibacterial activities of the *A. coriaria* leaves. For example, alkaloids, saponins, tannins, and polyphenols (flavonoids and phenols) have reported antibacterial activities [110] which are attributed to both their direct action against microorganisms and suppression of microbial virulence factors [111]. Alkaloids function by penetrating cells, intercalating microbial DNA, and targeting several nucleic acid enzymes, resulting in irreversible damage to microbial cells [112]. Tannins and saponins inhibit microbial growth through precipitation of microbial proteins, rendering such nutritional proteins unavailable to the microorganisms [113]. Tannins may also disrupt bacterial enzymes, cell envelope, adhesions, and transport proteins. Their high affinity for iron in microbial cell membranes inactivates membrane-bound proteins, making extracts of gallotannin-rich plants exhibit antibacterial activities [114]. The observed antibacterial activities also correlate with the quantities of the secondary metabolites identified in the extracts. For example, ethanolic extracts had higher quantities of phenols and saponins compared to ethyl acetate and aqueous extracts (Table 2). Ethanol extract of leaves from Mbarara which had the highest bioactivity had the highest concentration of phenols, saponins, and tannins, in addition to alkaloids that were not detected in other ethanolic extracts.

#### 3.4.2. Minimum Inhibitory Concentration (MIC) and Minimum Bactericidal Concentration (MBC) of Ethanol Extracts of *A. coriaria* Leaves

Ethanolic extracts were the most active and were thus considered for further tests for MIC and MBC against *S. aureus*, *P. aeruginosa*, and *S. typhi*. Ethanol extract of Mbarara leaves had the lowest MIC of 62.5 *µ*g/mL on *P. aeruginosa* followed by *S. aureus* with MIC of 125 *µ*g/mL (Table 7). This showed that the extract was more effective against *P. aeruginosa* compared to *S. typhi* and *S. aureus.* A previous study utilizing ethyl acetate extract of *A. coriaria* stem bark indicated a higher MIC of 125 mg/mL (125,000 *µ*g/mL) for *E. coli* and 250 mg/mL (250,000 *µ*g/mL) for *P. aeruginosa* [58]. In another investigation, ethanolic extracts of *A. coriaria* stem bark had MIC greater than 256 *μ*g/mL for *P. aeruginosa* AH-71 [11]. The authors further reported that ethanol and ethyl acetate extracts of *A. coriaria* stem bark had MIC of 250, 500, and > 500 *μ*g/mL against *S. aureus* ATCC 25923, *E. coli* K12 ATCC 23716, and *Listeria innocua* ATCC 33090 [61] which are comparable to the values obtained in this study.

The MBC of 125 *µ*g/mL of the extracts against *P. aeruginosa* showed that the extract has a stronger activity towards *P. aeruginosa* than the rest of the bacteria. A contrastingly higher MBC of 125 mg/mL for *E. coli* was reported for ethyl acetate extract of *A. coriaria* stem bark extract, while *P. aeruginosa* was not affected at the tested concentrations of 62.5, 125, 250, and 500 mg/mL [58]. Overall, previous studies using single-solvent extraction systems reported higher MIC and MBC values than those obtained in this study. For example, Luvonga [63] reported MIC of 12.5 mg/mL (1,250 *μ*g/mL) for *S. aureus* and 25.0 mg/mL (25000 *μ*g/mL) for *P. aeruginosa* when they were treated with aqueous extracts of *A. coriaria* stem bark. The corresponding MBC values were 12.5 mg/mL (12,500 *μ*g/mL) and 50.0 mg/mL (50,000 *μ*g/mL). Nalubega et al. [62] reported MIC of 0.5 g/mL (500,000 *μ*g/mL) for aqueous *A. coriaria* stem bark extracts on *S. aureus*. It should be emphasized that high MIC and MBC values are indicative that higher doses of the plant extracts are required for effective treatment. These results could explain why the posology of 3–4 cups per day of *A. coriaria* extracts is recommended by Ugandan traditional healers for adults [77].

## 4. Conclusion

This study for the first time established that *A. coriaria* leaves possess therapeutic phytochemicals with significant antioxidant and antibacterial activities, which lends credence to its use in traditional management of oxidative stress-induced conditions and bacterial diseases in Uganda. Ethanol was the best extraction solvent and should be used for further studies on the leaves of this species. The leaves of *A. coriaria* displayed intraspecific variation of secondary metabolites, total phenolic content, total flavonoid content, and antioxidant and antibacterial activities for the same solvent extracts of samples taken from different agroecological zones of Uganda. These variations could be due to differences in soil chemistry, rainfall, topography, and climate of the different agroecological zones which affected the interaction between the plants and the environment and the quantity of bioactive compounds in the *A. coriaria* leaves. The recorded phenolic and flavonoid contents as well as antioxidant and antibacterial bioactivities of the leaves are probably due to the secondary metabolites (alkaloids, phenols, saponins, flavonoids, cardiac glycosides, tannins, and terpenes) identified in the extracts. Toxicity studies of the leaf extracts are recommended, so as to establish their safety when utilized as crude extracts in traditional medicine. An extension of this study is characterizing the specific compounds in the ethanolic extracts of leaves from the different districts using a combination of chromatographic and spectroscopic analytical techniques.

## Figures and Tables

**Table 1:** Location of *A. coriaria* trees where leaves were sampled in Uganda.

District	Location	Geographical position system location
Jinja	Along Kyabazinga Way (near AgroWays Uganda Limited)	0°26′34.3″N 33°12′27.3″E
Kole	Otangula village, Ilera Parish	2°17′35.0″N 32°46′31.2″E
Mbarara	Ruharo ward, Ruhizi cell, Kamukuzi division	0.6164° S 30.6186° E

**Table 2:** Secondary metabolites identified in *A. coriaria* leaves from Uganda.

Phytochemicals	Extract	Jinja	Kole	Mbarara
Alkaloids	Ethyl acetate	++	++	−
	Ethanol	−	−	++
	Aqueous	+	+	+

Cardiac glycosides	Ethyl acetate	++	+	++
	Ethanol	+	+	+
	Aqueous	+	+	+

Flavonoids	Ethyl acetate	−	+	++
	Ethanol	+	++	++
	Aqueous	++	++	++

Phenols	Ethyl acetate	++	++	++
	Ethanol	++	++	+++
	Aqueous	+	+	+

Quinones	Ethyl acetate	−	−	−
	Ethanol	−	−	−
	Aqueous	−	−	−

Saponins	Ethyl acetate	+	+	+
	Ethanol	++	++	+++
	Aqueous	+	+	+

Steroids	Ethyl acetate	−	−	−
	Ethanol	−	−	−
	Aqueous	−	−	−

Tannins	Ethyl acetate	+	+	+
	Ethanol	−	+	+++
	Aqueous	++	++	++

Terpenes	Ethyl acetate	++	++	+
	Ethanol	−	++	+++
	Aqueous	+	+	+

Volatile oils	Ethyl acetate	−	−	−
	Ethanol	−	−	−
	Aqueous	−	−	−

Note: +++ represents very high, ++ indicates moderate, + indicates little/traces, and − indicates absent.

**Table 3:** Total phenolic content of the *A. coriaria* leaves from Jinja, Kole, and Mbarara districts of Uganda.

District	Ethyl acetate extract (mg GAE/g DW)	Ethanolic extract (mg GAE/g DW)	Aqueous extract (mg GAE/g DW)
Jinja	16.88 ± 0.11^a^	67.04 ± 0.19^b^	5.29 ± 0.13^c^
Kole	10.93 ± 0.13^a^	77.99 ± 0.17^b^	20.69 ± 0.27^c^
Mbarara	60.69 ± 0.23^a^	101.72 ± 0.22^b^	61.25 ± 0.13^a^

Different superscript letters indicate statistical difference at *p* < 0.05 between the solvent extracts.

**Table 4:** Total flavonoid content of *A. coriaria* leaves from Jinja, Kole, and Mbarara districts of Uganda.

District	Ethyl acetate extract (mg QE/g DW)	Ethanolic extract (mg QE/g DW)	Aqueous extract (mg QE/g DW)
Jinja	0.55 ± 0.01^a^	8.63 ± 0.02^b^	2.74 ± 0.02^c^
Kole	2.50 ± 0.04^a^	11.58 ± 0.04^b^	2.35 ± 0.05^a^
Mbarara	9.66 ± 0.01^a^	13.23 ± 0.03^b^	3.36 ± 0.04^c^

Different superscript letters in a row indicate statistical difference at *p* < 0.05 between the solvent extracts.

**Table 5:** DPPH results for inhibition concentration at 50% (IC_50_) of leaves from Jinja, Kole, and Mbarara districts of Uganda.

District	Ethyl acetate extract (mg/mL)	Ethanolic extract (mg/mL)	Aqueous extract (mg/mL)
Jinja	23.99 ± 0.05^a^	23.41 ± 0.13^a^	29.80 ± 0.26^a^
Kole	26.34 ± 0.09^b^	23.18 ± 0.09^a^	29.66 ± 0.21^a^
Mbarara	23.73 ± 0.16^a^	18.65 ± 0.06^b^	25.51 ± 0.14^b^

Different superscript letters in a column indicate statistical difference at *p* < 0.05 between the different solvent extracts.

**Table 6:** Zone of inhibition of *A. coriaria* extracts against the tested pathogenic bacteria.

District	Extract	*E. coli* (mm)	*S. aureus* (mm)	*P. aeruginosa* (mm)	*S. typhi* (mm)
Jinja	Ethyl acetate	0.00 ± 0.00	0.00 ± 0.00	0.00 ± 0.00	0.00 ± 0.00
	Ethanol	6.00 ± 1.73	5.00 ± 1.00	18.00 ± 2.65	9.00 ± 1.73
	Aqueous	0.00 ± 0.00	0.00 ± 0.00	0.00 ± 0.00	0.00 ± 0.00

Kole	Ethyl acetate	3.00 ± 0.00	0.00 ± 0.00	0.00 ± 0.00	0.00 ± 0.00
	Ethanol	7.00 ± 1.00	6.00 ± 0.00	17.00 ± 0.00	16.00 ± 1.73
	Aqueous	0.00 ± 0.00	0.00 ± 0.00	0.00 ± 0.00	0.00 ± 0.00

Mbarara	Ethyl acetate	4.00 ± 2.00	0.00 ± 0.00	0.00 ± 0.00	0.00 ± 0.00
	Ethanol	10.00 ± 1.73	12.30 ± 1.53	25.00 ± 2.65	10.00 ± 0.00
	Aqueous	0.00 ± 0.00	0.00 ± 0.00	0.00 ± 0.00	0.00 ± 0.00

Ciprofloxacin		14.00 ± 2.10	12.00 ± 0.01	31.00 ± 0.11	20.00 ± 1.53
Dimethyl sulfoxide		0.00 ± 0.00	0.00 ± 0.00	0.00 ± 0.00	0.00 ± 0.00

Results are presented as mean ± standard deviation of triplicates.

**Table 7:** Minimum inhibitory and minimum bactericidal concentrations of the ethanol extracts of *A. coriaria* leaves against the susceptible tested bacteria.

District	MIC (*µ*g/mL)			MBC (*µ*g/mL)		
	*S. aureus*	*P. aeruginosa*	*S. typhi*	*S. aureus*	*P. aeruginosa*	*S. typhi*
Jinja	ND^*∗*^	125	ND	ND	250	ND
Kole	ND	250	250	ND	250	250
Mbarara	125	62.5	ND	250	125	ND

^*∗*^ND: not determined as bacteria were not susceptible to the extract. Values reported are from assays performed in triplicate.

## Data Availability

The datasets supporting the conclusions of this study are available from the corresponding author upon request.
